# Targeting Mitochondrial Singlet Oxygen Dynamics Offers New Perspectives for Effective Metabolic Therapies of Cancer

**DOI:** 10.3389/fonc.2020.573399

**Published:** 2020-09-18

**Authors:** Jorgelindo da Veiga Moreira, Laurent Schwartz, Mario Jolicoeur

**Affiliations:** ^1^Research Laboratory in Applied Metabolic Engineering, Department of Chemical Engineering, Polytechnique Montréal, Montréal, QC, Canada; ^2^Assistance Publique des Hôpitaux de Paris, Paris, France

**Keywords:** cancer, mitochondria, singlet oxygen therapy, chemoresistance, metabolic therapy

## Abstract

The occurrence of mitochondrial respiration has allowed evolution toward more complex and advanced life forms. However, its dysfunction is now also seen as the most probable cause of one of the biggest scourges in human health, cancer. Conventional cancer treatments such as chemotherapy, which mainly focus on disrupting the cell division process, have shown being effective in the attenuation of various cancers but also showing significant limits as well as serious sides effects. Indeed, the idea that cancer is a metabolic disease with mitochondria as the central site of the pathology is now emerging, and we provide here a review supporting this “novel” hypothesis re-actualizing past century Otto Warburg's thoughts. Our conclusion, while integrating literature, is that mitochondrial activity and, in particular, the activity of cytochrome c oxidase, complex IV of the ETC, plays a fundamental role in the effectiveness or non-effectiveness of chemotherapy, immunotherapy and probably radiotherapy treatments. We therefore propose that cancer cells mitochondrial singlet oxygen (^1^O_2_) dynamics may be an efficient target for metabolic therapy development.

## Introduction

Oxygen is “the molecule that made the world” stated Lane in his seminal book (1). Nowadays, the atmosphere of Earth is composed of 21% oxygen and used by most organisms for respiration (animals, plants, and many prokaryotes). These living organisms use oxygen as oxidizing agents to retrieve energy from reduced compounds (2).

Human body is made up of 10,000–100,000 billion cells. Every day tens thousands of cancer cells are formed but are also soon eliminated by the immune system (3). The other common point between cancer cells and pathogenic organisms is their dependence on metabolism of healthy cells and the irrigation of nutrients by the blood system to ensure their replication (4). In this way, competition arises between cancer cells and somatic cells for access to nutrients, specially to glucose, and oxygen. This competition is similar to game theory as introduced by John von Neumann (5) then applied to biology by John Maynard Smith (6). Transposed to game theory, the metabolism of healthy cells and cancer cells are distinguished by the way they produce energy in the form of ATP. In presence of oxygen, healthy cells use glucose to produce about 30 molecules of mitochondrial oxidative phosphorylation ATP. Under hypoxic conditions, they produce only 2 molecules of ATP through glycolysis and release 2 molecules of lactic acid. As far as cancer cells are concerned, they favor the glycolysis pathway for energy production, even in oxygenated environment, referred as aerobic glycolysis or “Warburg effect” named after the German physician and biochemist Otto Heinrich Warburg who first reported this observation (7–9). Warburg showed that cancer cells in culture have higher rates of glucose consumption and lactate secretion compared to normal cells and hypothesized mitochondrial dysfunction to explain this glycolytic phenotype. However, many more recent studies have demonstrated the integrity of mitochondria in cancer cells (10, 11).

Here, we bring an overview of the common therapeutic approaches such as chemotherapy to circumvent tumor progression and limits thereof. We specially focused on the involvement of mitochondria regarding the metabolic adaptation of cancer cells to escape from apoptosis and promote, in some way, cancer recurrence (12). In perspective of this study, we propose that the cancer metabolic phenotype or Warburg effect could be relieved by the controlled generation of mitochondrial singlet oxygen (^1^O_2_). Singlet oxygen is the first excited state of the dioxygen molecule and is part of the ROS species generated during the OXPHOS process. We support the idea that the generation of ^1^O_2_ in the respiratory chain is a necessary step for OXPHOS and ATP production. Our hypothesis is that the mitochondrial accumulation of ROS observed during phases of high energy demand or in periods of substrates deficiency would be due to a limitation of the respiratory capacity of cells in general, and in particular in tumors where physical and metabolic variabilities have been reported (13). This, is a fundamental review deciphering on how cancer cells' ROS production is an adaptation to escape apoptosis and how strategies such as singlet oxygen-oriented therapy could potentially counteract cell proliferation and metastasis.

## Mitochondrial Respiration is the Key to Understand Cancer Metabolism

The cell cycle is also a metabolic cycle regulated by redox transitions promoting fermentation or cellular respiration (14). Cancerous masses deploy a panoply of adaptative actions in the face of external stresses such as chemotherapy. This is partly made possible by the “hijacking” of the genetic program for regulating the cell cycle. In the following, we will thus first describe the mitochondrial metabolism and the electron transfer chain (ETC) functioning. Then we will decipher how cancer cells manage to “hijack” mitochondrial activity in its favor and to escape from apoptosis even under ROS-induced chemotherapy treatments.

### Energetic Metabolism and the Respiratory Chain

Mitochondria are involved in various cellular processes such as differentiation and cell death (apoptosis) as well as supporting the immune response (15, 16). The mitochondria are the energy factories in eukaryotes. They mobilize the enzymes necessary for the proper functioning of the citric acid cycle (TCA, or Krebs cycle) and are involved in the management of the redox balance. Mitochondria use oxygen to extract energy from carbon-based nutrients found in the cellular environment or stored as intracellular macromolecules like glycogen and results in the synthesis/recycling of adenosine triphosphate (ATP) by phosphorylation of adenosine diphosphate (ADP). This process called oxidative phosphorylation (OxPhos) takes place at the level of the mitochondrial ETC chain.

### The “Classical” Respiratory Chain of Eukaryotic Cells

**Figure 1:**
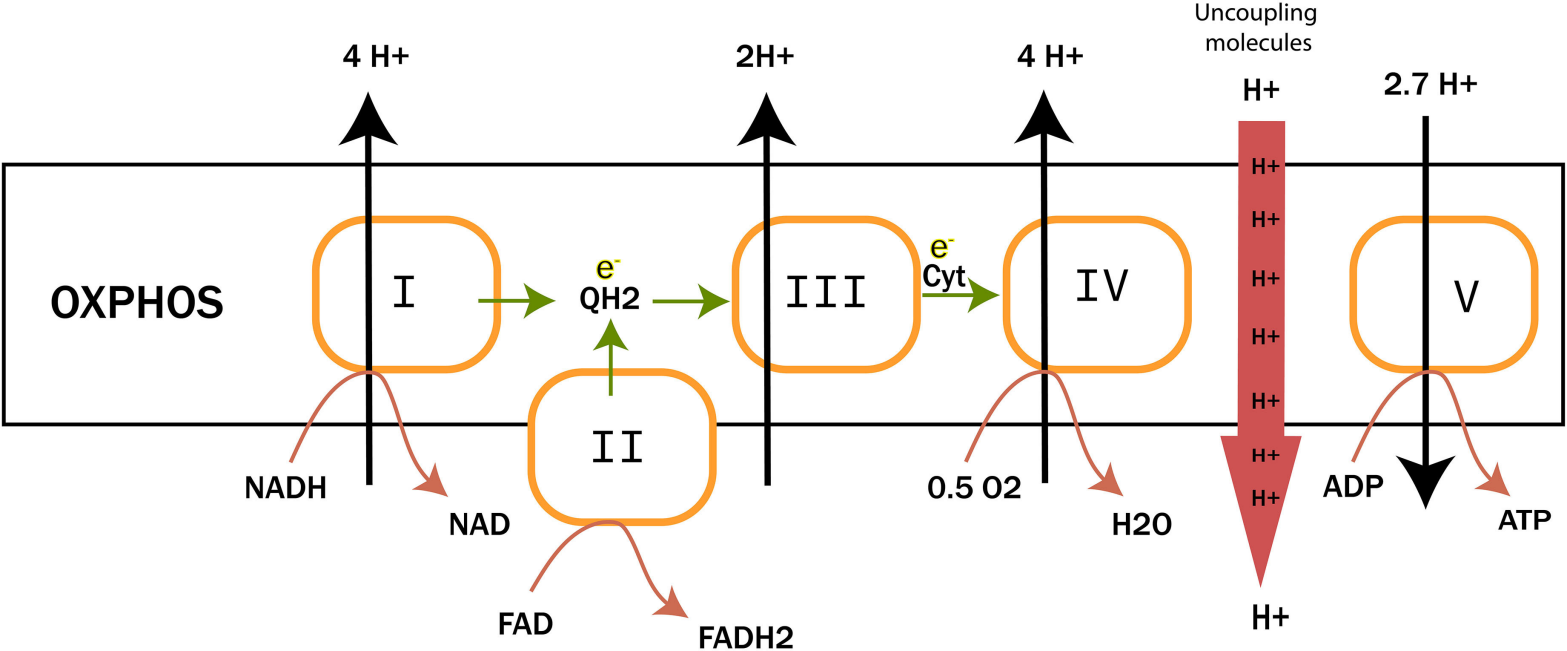
The electron transfer chain (ETC). It is composed of four complexes involved in electron transfer and proton translocation. Complex V or ATP synthase catalyzes ATP synthesis. Uncoupling proteins and molecules are also reported to trigger proton leaks to mitochondrial matrix.

Peter D. Mitchell proposed in 1961 the chemiosmotic theory to explain the synthesis of ATP by OxPhos mechanism. This theory suggests that the production of ATP is made possible by a proton gradient (ΔpH) formed on either sides of the inner membrane of the mitochondria thanks to an ATPase catalyzing the reaction (17). The proteins animating this respiratory chain promote the creation of this ΔpH thanks to the energy of the redox couples (NAD^+^/NADH and FAD/FADH_2_) present in the mitochondrial matrix and brought about by the catabolism of carbon-based resources. ATPase takes advantage of this electrochemical gradient also called protonmotive force (Δp) for the phosphorylation of ADP into ATP. The ETC of eukaryotic cells is essentially composed of enzymes and co-enzymes involved in the transfer of electrons and the synthesis of ATP. Four protein complexes (CI-IV) are involved in electron transport and oxygen reduction. ATP synthase, also known as complex V (CV), catalyzes ATP synthesis (Figure 1) (1801). A special focus is placed on complex IV (IV) or cytochrome c oxidase, since this enzyme has a pivotal role in mitochondrial respiration. It catalyzes the transfer of electrons from the reduced form of cytochrome c to dioxygen, which becomes the final acceptor of electrons in the respiratory chain (19, 20). Two molecules of water are formed through this reaction and four protons are pumped to the intermembrane space. This enzyme has been well-studied, in particular for understanding the mechanisms allowing both the reduction of dioxygen into water molecules and the flow of protons through the protein structure (21). The pivotal role of prosthetic (22) groups has been demonstrated. These metallic prosthetic groups form two copper redox centers (CuA and CuB) and two heme centers (heme *a* and heme *a*_3_). CuB and heme a3 are physically close and form a bi-nuclear center where the oxidation of oxygen takes place. CuA is the first electron acceptor of cytochrome c while heme a serves as an intermediary for the transfer of electrons between CuA and the binuclear center for the reduction of O_2_ (18). A detailed model of the O_2_ activation and its reduction cycle has been proposed (23, 24).

### Regulated Steps of Mitochondrial Respiration

The desire to identify a major OXPHOS “controller” began in the 70–80s (25), notably thanks to contributions of the Metabolic Control Theory (MCT) (26–28). CIV, CV, and the mitochondrial ATP/ADP translocase were the main candidates (29, 30). Subsequently, work on mitochondria isolated from liver cells has highlighted the role of these proteins, depending on the energy state of the cell (29). In short, during highly active OxPhos phase, the supply of electrons from substrates and the electron flux through the ETC were estimated as the limiting steps. During the stationary phase of respiration, proton translocation through mitochondrial membrane was the limiting step. Finally, when the cell's energy demand is reduced, the activity of ATP translocase became the limiting factor for OxPhos.

### The Potential Role of Apoproteins in Mitochondrial Mode Switch

ETC complexes behave like real biological capacitors, by analogy with electrical circuits. They have the capacity to store the electrons recovered from NADH or succinate. In 1964, John Rieske isolated a subunit of Complex III. He highlighted the presence of an iron-sulfur atomic aggregate on this apoprotein (31). This component of complex III has since become the Rieske protein while other proteins have been identified and assimilated to the Rieske protein due to the presence of the iron-sulfur cluster [Fe-S]. This is the case for complexes I and II of the respiratory chain (32, 33) but also for aconitase (34) which also consist of one or more several centers [Fe-S]. One may wonder if inhibition of these clusters could impair cellular respiration. Few studies have been devoted to this problem. Graham and Trumpowe (35) carried out mutations in the conserved domains of the Rieske protein in the yeast *Saccharomyces cerevisiae*. They observed that directed-mutations on codons of amino acids known to interact specifically with the [2Fe-2S] cluster and present exclusively in aerobic organisms lead to an inability of these mutants to use non-fermentable carbon sources for their growth (35). As described above, this prosthetic group, which is also present in cytochrome *bc1* (complex III) is the first electron acceptor emitted by ubiquinol (Q) and participates in the creation of the protonmotive force. Other studies emphasize the importance of the [2Fe-2S] cluster in maintaining OxPhos. Diaz et al. (36) have notably demonstrated that mutations on the gene coding for Rieske protein in fibroblasts causes a decrease in the synthesis of complexes I and IV and OxPhos deficiency (36). These [Fe-S] clusters are not the only prosthetic groups to play a major role in mitochondrial respiration. The copper (CuA and CuB) and heme centers of the CIV, involved in electron transfer and O_2_ activation reduction (24, 37), have a pivotal role in maintaining the activity of the other complexes of the ETC (38). Likewise, it has been recently demonstrated that the activity of CIV is regulated by Hypoxia-induced Factor (HIF) proteins in yeasts and animal cells (39, 40). Interestingly, these proteins which belong to the HIF family, do not regulate the expression of CIV specifically but rather the formation of supercomplexes or respirasomes (41, 42). Importantly, HIF proteins are well-known to play an important role in the metabolic reprogramming, evading immune surveillance and resisting death of cancer cells (43–45). HIF-1 signaling pathway allow cancer cells to adapt to the use of a particular substrate or to adapt to environmental stresses by optimizing the flow of electrons through ETC and escape programmed cell death (43, 45, 46).

## Current Cancer Treatments: Efficiency and Limits

Biochemical approaches to the management of cancer patients represent most treatments. They mainly include chemotherapy. However, in recent years immunotherapy treatments have become popularized, as well as metabolic therapy but to a lesser extent.

### Chemotherapy

Standard chemotherapy protocols for cancer combine therapeutic agents that induce DNA damage with another agent, usually from the taxane class, that inhibits microtubule dynamics (47, 48). An anti-angiogenic agent targeting neo-tumor vessels may be combined depending on the case. These chemotherapeutic agents are inoculated intravenously (IV). Intraperitoneal (IP) chemotherapy can also be used to deliver higher doses of chemotherapeutic agents to the peritoneal cavity, the metastatic site for ovarian cancer, for example. The main chemotherapeutic agents offered to patients are platinum salts, especially cisplatin. They belong to the class of alkylating compounds. They cause the proliferation of cells to stop after binding to DNA. The cytotoxic potential of cisplatin was incidentally discovered in 1965 by B. Rosenberg et al. When they applied an electric field from platinum electrodes to a culture of *Escherichia coli* bacteria, they noticed that cell division had stopped (49). They demonstrated that the inhibition of cell divisions was due to the formation of a complex between the platinum produced by the electrodes and the ammonium chloride in the medium. Since then, several platinum complexes have been tested to analyze their cytotoxic effect. Cis-dichloro-diamino-platinum (II), or CDDP, is the compound with the most pronounced effects (50).

**Figure 2:**
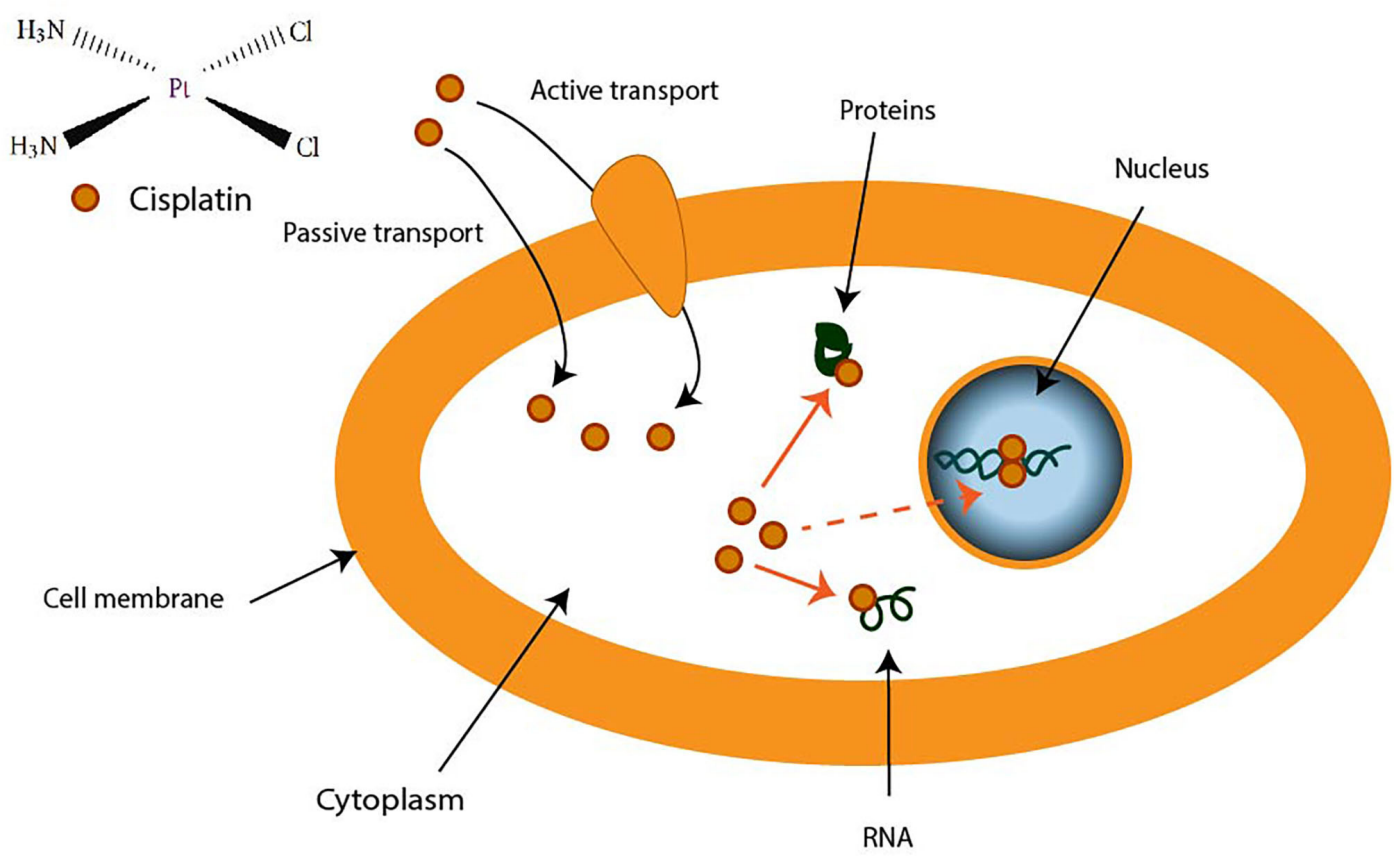
Transport of cisplatin to intracellular medium. Cisplatin crosses cell membrane by passive diffusion and by active transport. It binds to DNA, RNA, and some proteins.

CDDP is a molecule made up of a central platinum atom, of two labile chlorine atoms in the cis position and two inert ammonia groups (Figure 2). Rosenberg and VanCamp showed that the CDDP presented a significant anti-tumor activity in mice having developed sarcomas and leukemias (51). Subsequently, human clinical trials have shown the effectiveness of CDDP-based treatments for testicular cancer (52). Its use as an anti-tumor compound was validated by the FDA (Food and Drug Administration) in 1978. More than 35 years later, the CDDP remains the most used drug in the treatment of ovarian cancer. The CDDP enters the cell by passive diffusion, facilitated via copper or active transporters (53, 54). In the cytosol, the chloride ions (Cl^−^) of the CDDP are substituted by hydroxyl groups (^**.**^OH) (Figure 2). At physiological pH, these electrophilic complexes react with nucleophilic sites such as the nitrogenous bases of the DNA molecule. However, only 5–10% of the total concentration of intracellular CDDP are found in the nucleus. The remaining CDDP fraction binds to RNAs, proteins, or glutathione (55). In the event that DNA damage becomes permanent, the signaling pathways that lead to cell death are activated (56). Thus, the ability of cells to make repairs following DNA damage caused by CDDP modulates their sensitivity to treatments. Cisplatin is the first platinum derivative to be used in chemotherapy. However, its high toxicity limits its use (nephrotoxicity, neurotoxicity, ototoxicity). Three analogs can be used in chemotherapy: carboplatin, oxaliplatin, and nedaplatin. Often combined with platinum salts (cisplatin or carboplatin) for the treatment of ovarian cancer, taxanes (paclitaxel or docetaxel) are natural alkaloid diterpenes extracted from bark or yew needles (57, 58). Taxanes exert their cytotoxic effect by binding to microtubules in the cell cycle and inhibit cell division by preventing depolymerization of microtubules during G2/M phase (before mitosis) (59, 60). On the other hand, weekly administration of paclitaxel has been shown to induce cell death independent of microtubule stabilization. The transcription of different genes involved in repairing DNA damage, inflammation, or cell proliferation is, in fact, also modulated. In addition, several apoptotic or oxidative stress signaling pathways are also activated in response to paclitaxel. All these strategies are applied to potentiate the anti-cancer activity of paclitaxel.

### Immunotherapy

**Figure 3:**
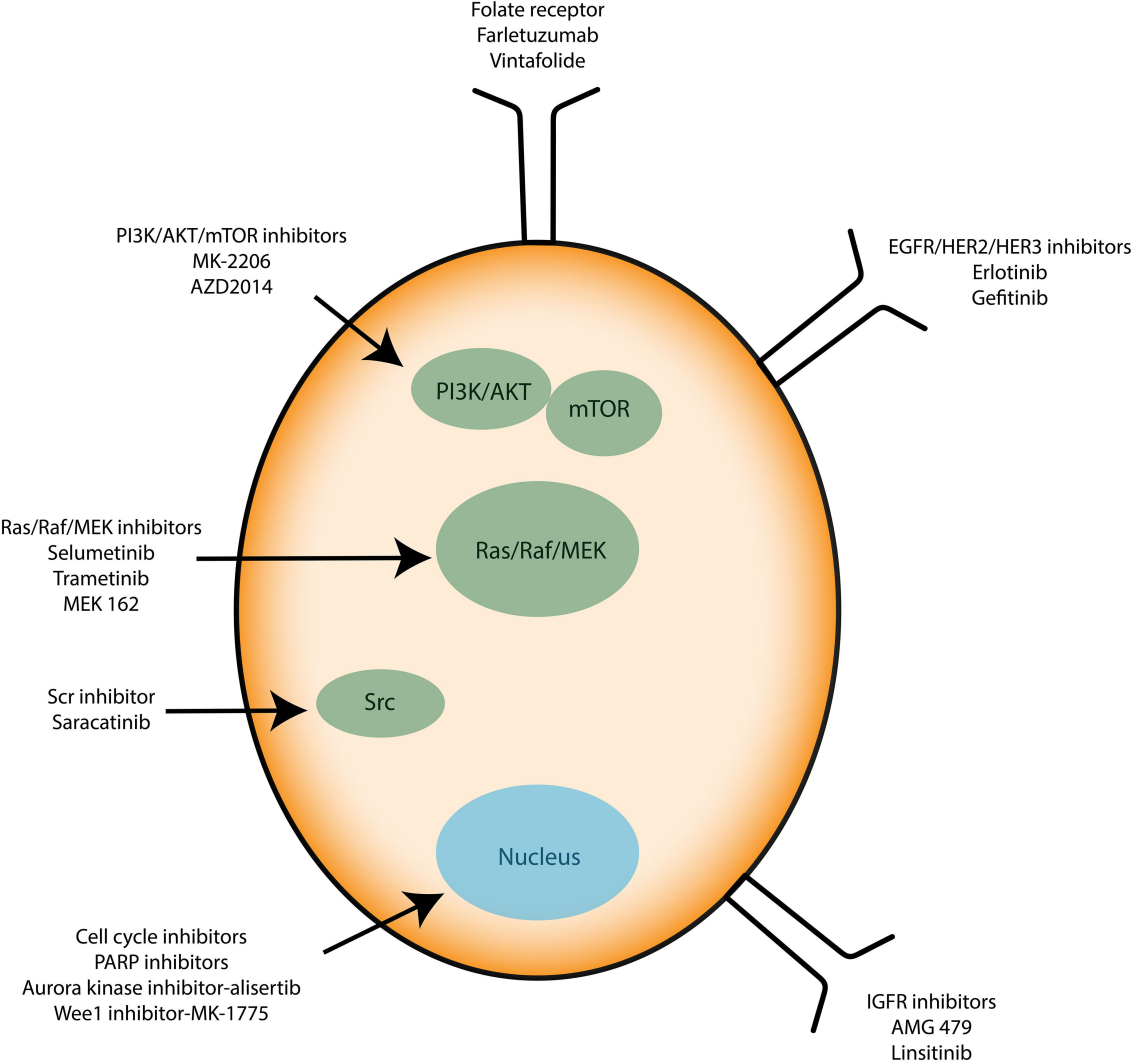
Immunotherapies applied to ovarian cancer cells. Known therapies mainly target signaling proteins involved in cell proliferation and resistance to apoptosis such as PI3K/AKT/mTOR, Src, and Ras/Raf/MEK. Growth factor receptors and cell cycle inhibitors are used too.

Recently, anti-angiogenic agents have been introduced into ovarian cancer treatment protocols. They target the formation of new vessels from preexisting vessels (angiogenesis), a process necessary for tumor survival and spread. Bevacizumab (Avastin®) is a monoclonal antibody that binds to the pro-angiogenic factor VEGF (Vascular Epidermal Growth Factor) and prevents its interaction with its receptors located on the surface of endothelial cells (61). In parallel, many clinical trials aim to use small inhibitory pharmacological molecules targeting the processes involved in tumor growth and/or spread (antibodies, inhibitors). Special mention of antibodies directed against pro-angiogenic factors (anti-VEGF antibodies), molecules blocking DNA repair systems (PARP inhibitor) or also inhibitors of folate receptors, the latter being overexpressed by ovarian cancer cells. Molecules that interfere with altered signaling pathways in ovarian cancer are also developed. They target the MAP-Kinases, PI3-Kinase/Akt pathways or integrin-type adhesion receptors. Immunotherapy strategies are also being considered (antibodies, inhibitors of immunological checkpoints, vaccines) (Figure 3). All these trials open up new therapeutic perspectives with the hope of seeing new molecules coming to the market for the treatment of ovarian or other type of cancer such as glioblastoma multiforme (GBM) (62).

## Cancer Recurrence: the Special Case of GBM and Ovarian Cancer

In the case of ovarian cancer, 70–80% of patients respond well to initial treatments. However, more than 80% of patients relapse within the next 6–36 months. Second line treatments include a cytoreductive surgery combined with chemotherapy (63). Second-line treatments aim to improve the quality of life of patients and increase the progression-free survival of the disease. In the absence of effective therapies, most recurrent ovarian cancers are incurable. This is likely due to phenotypic plasticity and adaptation, a process common to cancer cells (64, 65). During the recurrence process, ovarian cancer cells acquire an adaptive phenotype allowing them to overcome the mechanisms guarantying cell and tissue homeostasis. These characteristics were identified in 2000 and supplemented in 2011 by Hanahan and Weinberg (66, 67). These characteristics are: (i) self-sufficiency in proliferative signals (ii) insensitivity to growth inhibition signals, (iii) ability to replicate indefinitely, (iv) genomic instability, (v) angiogenic induction potential, (vi) an ability to invade tissues and form metastases, (vii) a misregulation of cellular energy metabolism, (viii) an ability to escape the immune system and to promote an inflammatory environment, and (ix) the ability to resist cell death. This constitutes a major obstacle in the therapeutic management of cancers. Taken all the above, a special focus need being placed on points (I and II), and to decipher signaling pathways that could explain the phenotypic variability within a tumor mass leading to resistance to programmed cell death.

### Chemoresistance of Ovarian Cancers

Chemoresistance is defined as the ability of cancer cells to survive the cytotoxic effect of anticancer agents. It can manifest itself right away, i.e., intrinsic chemoresistance. In other cases, the treatments make it possible to obtain a partial or incomplete response, which then become ineffective over time. This is defined as acquired chemoresistance. Loss of sensitivity of ovarian cancer cells to platinum-based treatments is one of the major complications of ovarian cancer. The mechanisms leading to the appearance of this chemoresistance have been classified in four categories according to the moment when they intervene in the response chain of the CDDP (68). Two mechanisms promote the inhibition of the cytotoxic effect of CDDP before its interaction with DNA. A decrease in the concentration of intracellular CDDP is observed following an inhibition of facilitated diffusion by the copper transporter or an increase in its efflux by ABC-type transporters (ATP Binding Cassette). For example, overexpression of ATP7A (a member of the ABC protein family) is associated with a poor prognosis (69). An increase in the sequestration of CDDP by thiols intracellular occurs. Indeed, glutathione (GSH) can react with CDDP thus forming a platinum-GSH complex which will be subsequently eliminated. γ-Glutamylcyteine synthetase, the enzyme responsible for the synthesis of GSH, has been shown to be overexpressed in ovarian cancers (70–72).

### Chemoresistance of Glioblastoma Multiforme

**Figure 4:**
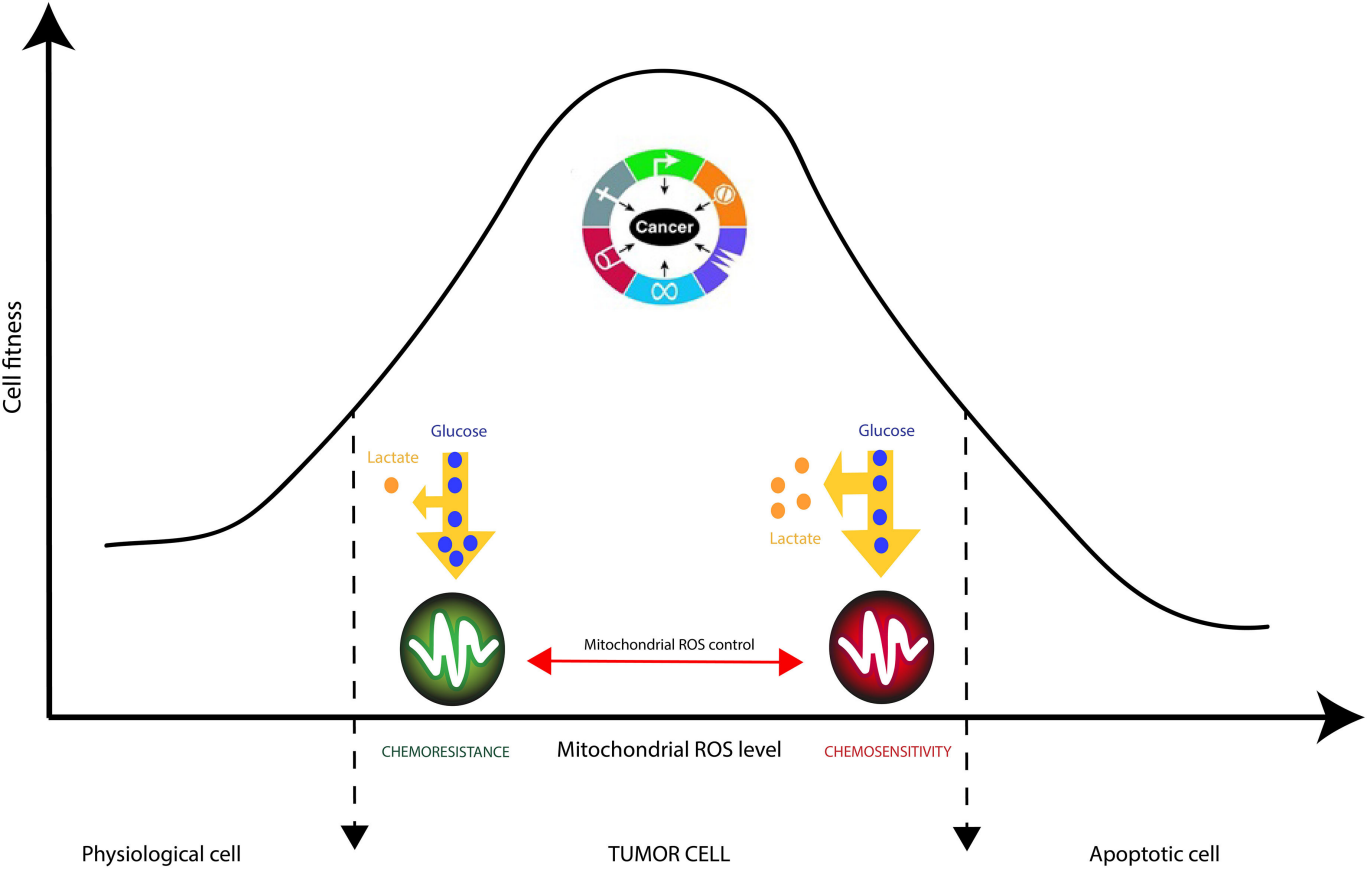
Mitochondrial checking of Reactive Oxygen Species (ROS) in cancer cells is crucial in tumor resistance to therapies. ROS accumulation is known to trigger cancer cell proliferation supported by nutrients consumption, manly glucose, and pronounced Warburg effect (increased lactate production). Strategic reduction of mitochondrial ROS by complex IV increases cancer cells' robustness to external stresses such as chemotherapies.

It is generally accepted that cancer cells produce large amounts of ROS, mainly in the inner mitochondrial membrane (IMM), the site of cellular respiration (73). When the flow of electrons is slowed down at the level of the electron transfer chain (ETC), this produces mitochondrial ROS (mtROS) species such as superoxide radical (O_2_^**.−**^), hydrogen peroxide (H_2_O_2_) or hydroxyl radical (^**.**^OH). These reactive species can, in the long run, lead uncontrolled oxidations of mitochondrial DNA (mtDNA), lipids and proteins. Interestingly, cancer cells have been shown to decrease the generation of mitochondrial ROS by improving their mitochondria coupling (74). This phenomenon even occurs when external ROS generation methods are applied. This is the case of glioblastoma multiforme (GBM) cells resistant to Temozolomide (TMZ), a chemotherapy drug commonly used for treating gliomas (75). Recent studies have demonstrated that the resistance of glioma cells to drugs are acquired characteristics that are linked to mitochondrial activity in general, and to the activity of complexes (I-IV) of the electron transfer chain in particular (13, 74–76). Oliva et al. (75) studied chemoresistance in a glioma cell line and xenograft using TMZ chemotherapy agent. They managed to show that TMZ-resistant cells have a better management of mtROS generation due to a higher mitochondrial coupling. Moreover, the chemoresistant glioma cell line showed reduced mtROS production concomitant with increased oxygen consumption, and lowered proton leak. Interestingly, the authors showed that TMZ-resistant cells consumed less glucose and produced less lactate which are markers of reduced Warburg effect (Figure 4). Finally, the authors managed to reserve TMZ resistance and increased sensitivity to chemotherapy by treating glioma cells with the oxidant L-buthionine-S,R-sulfoximine. On the other hand, the antioxidant N-acetyl-cysteine treatment prevented TMZ cytotoxicity in sensitive cell lines by vanishing TMZ-dependent mtROS generation (75). These observations show the pivotal role of mtROS in the induction of pro and antitumor signaling pathways (74).

TMZ triggers mtDNA alterations and creates local mtROS accumulation. Then, proteins and lipids are at the mercy of mtROS, which trigger its oxidation and compromise mitochondrial membrane integrity. Therefore, mtROS seems to have a feed-forward loop effect on mtDNA. Indeed, it has been reported that minor mtDNA alterations and mutations are major contributors to mtROS accumulation in cancer cells (77). Exactly 13 ETC proteins are encoded in mtDNA and the rest are nuclear genome encoded (78). Mutations in the genes encoding these subunits cause ETC dysfunction and accumulation of mtROS (74, 79). Interestingly, Oliva et al. (75) also reported a sharp drop of TMZ-induced ROS generation in mitochondrial DNA-depleted (ρ°) glioma cell line and decreased cell sensitivity to TMZ drug. Sensitivity of the glioma cell line to TMZ was restored upon repopulating cell with functioning mitochondria. As we postulated above, mitochondrial metabolism and its integrity appear to play a pivotal role in the treatment of cancer and recurrences that may occur.

## Metabolic Therapies for Cancer Treatment

One of the most studied pathology related to mitochondrial metabolism is cancer. First studies date from the 1920s in which Otto Heinrich Warburg, future Nobel Prize of medicine winner in 1931, observes that cancer cells produce abnormally high amounts of lactate, even in oxygen aerated environment (9, 71, 80). He explained that cancer cells derive their energy mainly from the fermentation of glucose or aerobic glycolysis. Warburg later hypothesized that the fermentative phenotype of cancer cells was due to dysfunction of the mitochondria (8). Our recent works has helped into validation of some Warburg's assumptions. Indeed, we have demonstrated that human and mouse cancer cell lines have very low mitochondrial membrane potential (ΔΨm) and more pronounced glycolysis compared to the respective healthy cells (81). We also proposed complementary therapeutic approaches to chemotherapy, aiming to counteract the Warburg effect in cancer cells (34, 82–84). These are METABLOC, a combination of small molecules composed of α-lipoic acid, pushing carbon flux to mitochondria, and hydroxycitrate, an inhibitor of lipogenesis.

### Cancer Cells Have a Reduced Mitochondria Horsepower

Cancer cell growth is promoted by the anabolic signaling pathways and sustained metabolic reprogramming (45, 85, 86). We sought to establish the metabolic profile of cancer cells based on the characterization of physico-chemical parameters in healthy and cancerous primary cells isolated from the colon in patients. For each of the two populations, cells were separated by elutriation and then collected in different phases of the cell cycle (G0/G1/S/G2/M) according to their sizes. The parameters studied are the redox potential of the cells by quantification of the NA(D)/NAD(P)H species, the energetic state of the cells by ATP assay and the intracellular pH (pHi), which could be associated with the metabolic activity. The results confirm the two major phenomena associated with cancer cells: the metabolic activity is more pronounced compared to healthy cells, which results in a more alkaline pHi (81). Indeed, cancer populations in G0 have, on average, a pHi of 7.29 ± 0.13 while the pHi of healthy cells in the same phase of the cycle is 6.87 ± 0.10. We have also reported a lower energy efficiency in the cancer population. This is characterized by a lower amount of ATP (2X less in G_0_). The NAD^+^/NADH and NADP^+^/NADPH redox ratios are up to 5X and 10X higher, respectively, in cancer cells. It also reflects a more pronounced glycolytic flow as hypothesized by Warburg, while the production of lactate in the culture medium has not been determined. Indeed, a higher NAD^+^/NADH ratio is necessary for the maintenance of glycolysis while the production of lactate ensures the turnover of NAD^+^ by lactate dehydrogenase (LDH). Likewise, NADP^+^/NADPH conditions entry into the pentose phosphate pathway (PPP) and generates the NADPH necessary for the synthesis of fatty acids and of other membrane lipids. These results were then confirmed in various mice and human cell lines. In addition, ΔΨm has been quantified in healthy and cancerous cell lines. The results show that the mitochondrial membrane potential is significantly lower in cancer lines. This lower ΔΨm in cancer cells is assumed, such as lower energy efficiency (ATP synthesis), even when higher glycolytic flux is found in cancer cell population. Therefore, these results partially confirm Warburg's observations and offer avenues for therapeutic innovations.

### Metabolic Therapies Targeting Tumor Growth

**Figure 5:**
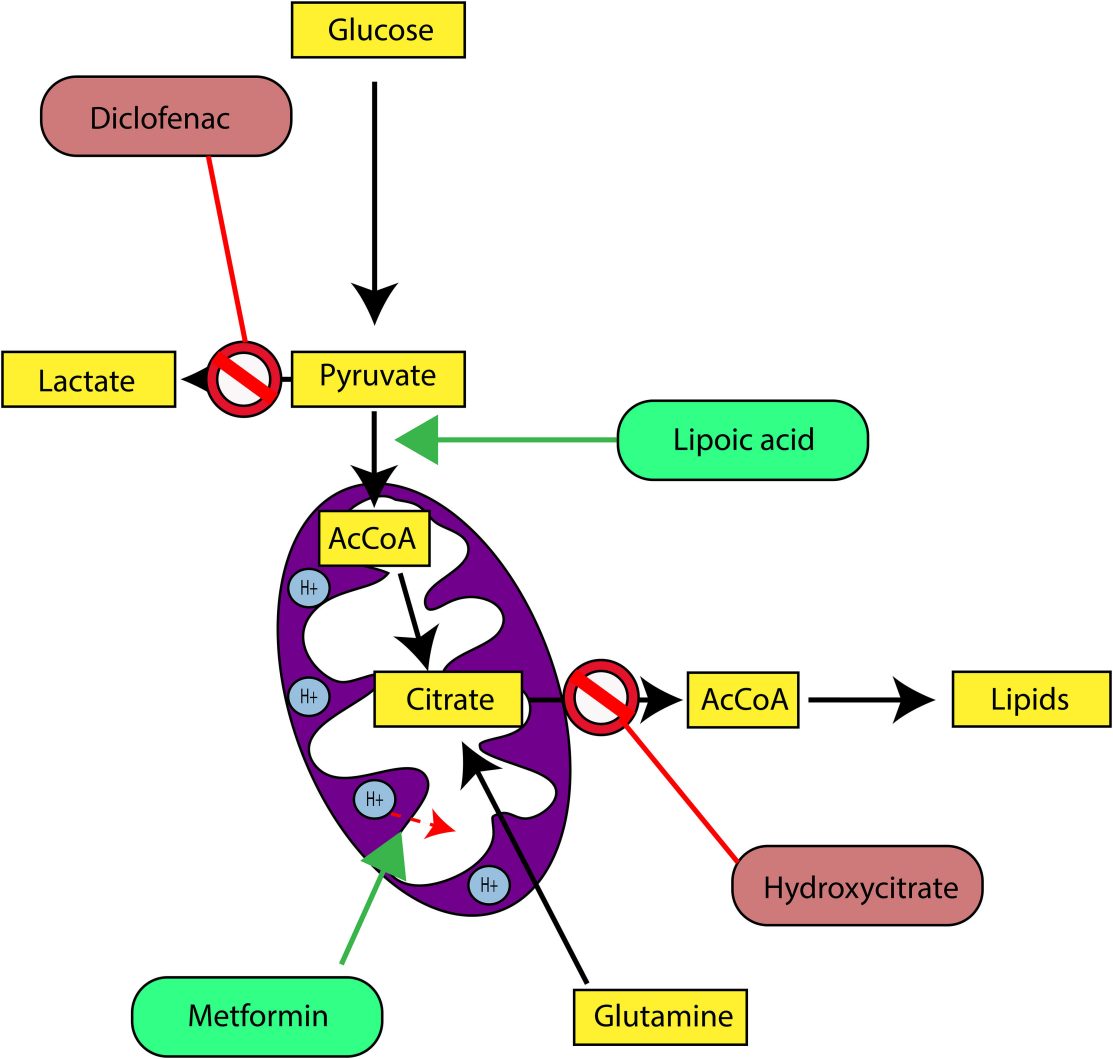
Cancer metabolic therapy targets central carbon metabolism and enhances mitochondrial activity. Diclofenac is an inhibitor of the Lactate dehydrogenase (LDH). By inhibition of dehydrogenase kinase-2 (PDK2), Lipoic acid promotes Pyruvate dehydrogenase (PDH) activity. Metformin activates mitochondrial uncoupling proteins and triggers mitochondrial membrane depolarization. Consequently, it increases electron transfer chain activities. Hydroxycitrate is an inhibitor of ATP-citrate lyase (ACL) and prevents cytosolic Acetyl-CoA (AcCoA) accumulation as precursor for lipogenesis.

In our last experimental study, we followed tumor growth in mice to which tumors were grafted and subjected to metabolic treatments for 59 days (84). The molecules used part of the METABLOC, supplemented with other molecules known from the pharmacopeia. These are α-lipoic acid (α-LA) and hydroxycitrate (HCA), both used as food supplements. The α-LA is an inhibitor of pyruvate dehydrogenase kinase-2 (PDK2), reported as inhibitor of pyruvate dehydrogenase (PDH) in normal cells but to a greater extent in cancer cell (85, 87, 88) (Figure 5). Interestingly, a recent study also reported effective inhibition of angiogenesis and HIF1-α activity in mice tumor xenograft under dichloroacetate (DCA) treatment (89), known as a major inhibitor of PDK2 (90). Moreover, PDK2 gene disruption in lung cancer cells have been reported to increase cell sensitivity to Paclitaxel chemotherapeutic agent (91). Part of METABLOC, HCA is an inhibitor of ATP-citrate lyase (ACL) to prevent lipogenesis (82, 92, 93). Finally, we used metformin, which is used in type II diabetes (94) and reported as inhibitor of complex I (95–97), and diclofenac, which is an anti-inflammatory and inhibitor of lactate dehydrogenase (LDH) and of the transporter of monocarboxylate (MCT1) (98, 99). A positive control group of mice was treated with cisplatin, a classic chemotherapeutic agent. We reported that the combination of these four molecules has an inhibitory effect on the growth of the tumor implanted in mice (84). Indeed, when the molecules are used separately, there are no major effects compared to the control group without treatment. However, when metformin is applied in high doses combined with α-LA and HCA, the growth of the tumor is clearly slowed down and then inhibited after ~50 days of follow-up. The inhibition is even more pronounced when high-dose diclofenac is added in combination with METABLOC and high-dose metformin.

In parallel with this experimental work, we have developed a kinetic model of central metabolism to simulate tumor growth and predict the effect of previous molecules on intracellular energy flows. This model includes a reduced and simplified metabolic network of cancer cell metabolism and manages to simulate tumor growth but also the inhibitory effect of the metabolic therapy. Simulations show a reverse Warburg effect under the action of metabolic therapy. This is evidenced by a net flow of blood lactate re-consumption through LDH and increased mitochondrial respiration, a characteristic phenotype of healthy cells. These experimental results show that it is possible to perturb the metabolic stability of cancer cells and restore a basal metabolism close to that of healthy cells. Mitochondrial metabolism in general, and cell respiration through the electron transfer chain in particular, therefore seems to be the ideal target to make tumor masses more vulnerable to treatments and for inducing apoptosis or necrosis of the tumor mass (84).

## Perspective: Over-Activating Cancer Cells Mitochondria by Singlet Oxygen-Oriented Photodynamic Therapy

As summarized in the previous sections, the synthesis of ATP by OXPHOS is made possible thanks to the successive transfers of electrons through the electron transfer chain (ETC) (Figure 1). Misregulation on one of these ETC complexes often causes metabolic disorders ranging from transient paralysis to prolonged degenerative processes such as myopathies, Alzheimer's, cancer, and other acquired degenerations (41). In the case of cancer, we have reported works showing the strict regulations of these complexes as well as the strategies adopted by cancer cells to escape from the immune system regulation. We bring here a new explanation, under strong assumptions, on the “mitochondrial dysfunctions” observed in certain sub-populations of cells as well as therapeutic solutions which could reverse the degenerative process linked to the proliferation and metastasis of most of tumor cells.

We propose that saturation of ETC with electrons could be the cause of the glycolytic phenotype of cancer cells but also of the accumulation of deleterious oxidizing species, such as ROS, also present in neurodegenerative pathologies (Alzheimer, Parkinson, Huntington). This electrochemical engorgement of the respiratory chain can as well be explained by the inhibition of complexes of the ETC which would prevent the transit of the electrons and by a modification of the physicochemical nature of oxygen (the final acceptor of the electrons in the ETC). Thus, cancer cells carry out a metabolic reprogramming to reach out a new metabolic steady state characterized by rapid proliferation and pronounced glycolysis, a specific phenotype described by DeBerardinis and Chandel (7) and Warburg (8). Our undisclosed preliminary results support the hypothesis of a modification of the energetic state of intracellular oxygen to explain an overvoltage at the “limits” of ETC.

### Oxygen Activation in Biological Systems

**Figure 6:**
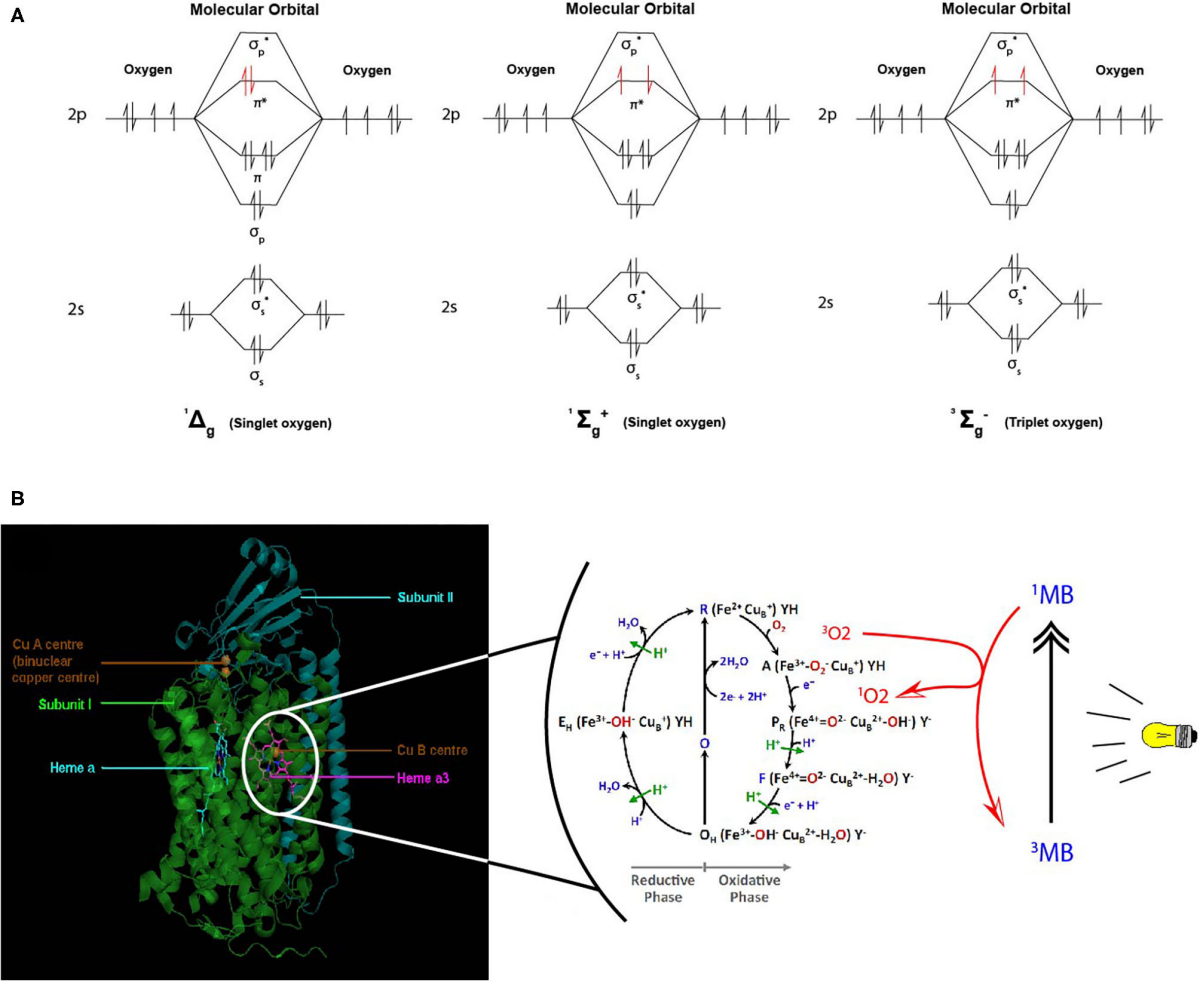
Proposed model of dioxygen reduction by cytochrome c oxidase (CcO) and potential role of combined light-methylene blue (MB)-induced singlet oxygen (^1^O_2_) generation. **(A)** Triplet and singlet oxygen molecular orbitals representation. Triplet oxygen (^3^∑) is the ground state, with unpaired spins at π^*^ orbital. Singlet oxygens (^1^Δ and ^1^∑) are the excited state with opposite spins at π* orbital. **(B)** The respiratory dioxygen reduction by CcO is ensured by the binuclear center with Heme a_3_ and Cu_B_. The catalytic cycle of O_2_ reduction to water molecules (H_2_O) is shown from R to E_H_ state. This cyclic model is proposed by Ishigami et al. (23). We obtained the kind permission of the corresponding author to use part of their figure initially in their study. In brief, the full cycle uses one O_2_ molecule, four electrons, four protons, and allows the pumping of additional four protons from the mitochondrial matrix to the intermembrane space. Two H_2_O molecules are produced. Electrons are sequentially transferred from the ETC cytochrome *c* Cu_A_ Heme *a*. Dioxygen first binds to heme *a*_3_ iron atom in reduced state (R) to form an intermediate state **(A)**. Then one electron is transferred from heme *a* to heme *a*_3_-iron and another one form Cu_B_ for the cleavage of O-O bonds. Follows sequential electrons and protons transfer until complete O_2_ reduction to water molecules. For more detailed description, see (23). We proposed that during the oxidative phase the oxygen molecules assume different oxidative forms with the iron atom which promote the production of ROS species (^1^O_2_,.OH,.${\text{O}}_{2}^{.-}$) during disturbances in the mitochondrial membrane due to toxic substances or instability of the lipid bilayer. Although the exact mechanism of formation of these ROS remains hypothetical, we propose a phototherapeutic approach consisting in the controlled generation of singlet oxygen via methylene blue (MB) or other photosensitizers. In this illustrating example, MB is in triplet (^3^MB) state in natural condition. When exposed to laser irradiation at specific wavelength (630–680 nm, <5 mW), ^3^MB reaches its excited ^1^MB state and triggers ^1^O_2_ generation. ^1^MB or other photosensitizers could be used for CcO-mimiking ^3^O_2_ activation and ^1^O_2_ generation in cancer cells. Mitochondrial singlet oxygen accumulation may rescue the Warburg phenotype and trigger cancer cell death.

Despites its high thermodynamic reactivity, dioxygen reacts slowly with most organic molecules because of spin restriction. Its stable state corresponds to a triplet electronic ground state, referred as triplet oxygen (^3^O_2_). The stable ^3^O_2_ has two unpaired electrons occupying the π molecular orbitals with the same spin orientation (Figure 6A). This prevents its spontaneous combustion with molecules having paired electrons (100). On the other hand, O_2_ can turn highly reactive with an input of energy and electronic excitation of the ground state ^3^O_2_. This excited state is referred as singlet oxygen (^1^O_2_) in which the electrons of the π orbitals are paired with opposite spin. This allows ^1^O_2_ to be much more reactive with organic molecules (101) (Figure 6A).

Generation of ^1^O_2_ from water molecule have been widely reported during photosynthesis in plants and cyanobacteria, using energy from the sunlight (102). In these systems, singlet oxygen is produced by light absorption by the photosensitizers. It is especially the case in plants where ^1^O_2_ is generated by the chlorophylls and other cofactors of the photosystem II (103). Once the highly reactive ^1^O_2_ is produced, it can be deactivated by quenching molecules such as beta-carotene, alpha-tocopherol, or plastoquinone. Cytochrome complex has also been involved in ^1^O_2_ production in plant (104). The authors showed that photoactivation of isolated cytochrome b6f complex triggers ^1^O_2_ generation. More precisely, it was shown the Rieske Fe-S protein-like cytochrome b6f center is the cluster involved in ^1^O_2_ production (31). Taken together, these observations raise the question of a possible involvement of singlet oxygen in mitochondrial respiration.

The eukaryotic respiratory chain has been extensively studied. As depicted in previous sections, it is essentially composed of five protein complexes involved in electron transfer, proton pumping across the mitochondrial membrane, oxygen reduction to water by the complex IV or cytochrome c oxidase (CcO), and ATP synthesis by ATP synthase (17, 25, 105–108). Three parameters have been proposed as key in controlling cell respiration: mitochondrial membrane pH gradient (ΔpH), O_2_ concentration and [ferricytochrome c]/[ferrocytochrome c] ratio at the ETC (109). Arnold et al. (109) first reported that CcO subunits stability is mediated by cardiolipin and essential for the regulations. They found that high mitochondrial matrix ATP-to-ADP ratio has an allosteric feedback inhibition on the complex IV and cell respiration. However, partial or total inhibition of the respiratory chain is one of the main causes of production of mitochondrial reactive oxygen species (ROS) (73, 74) and of actively-promoting mitochondrial metabolic switch supporting tumor progression and metastasis (13). Interestingly, singlet oxygen is reported as the main ROS produced during OXPHOS in yeast and human healthy and cancer cells' mitochondria (110). Its high reactive potential causes protein and mitochondrial DNA (mtDNA) damages (111, 112). However, new findings support the idea of metabolic and signaling activities of a mitochondrial low dose of ^1^O_2_ (113). Zhou et al. (113) triggered ^1^O_2_ generation in HeLa cancer cell line by laser irradiation and showed increased mtDNA replication, which is also a marker of increased mitochondrial respiration. Interestingly enough, pioneering studies showed that fast electron transfer through eukaryotes and prokaryotes electron transfer chain (ETC) achieves high O_2_ affinity to CcO (19, 21). More recently, studies have depicted the most probable mechanism beyond O_2_ activation by CcO complex (24, 37). CcO ensures cell respiration by sustaining both protons translocation and O_2_ reduction to water molecules. O_2_ activation is catalyzed by the binuclear heme-copper active site in a catalytic cycle by addition of four electrons routed through ETC (Figure 6B). Full O_2_ reduction is coupled with four protons translocation though the inner mitochondrial membrane (IMM) as showed in Figure 1 (20). The complete O_2_ reduction cycle model has been well depicted by (23), and the mitochondrial membrane stability seems to be a key parameter in oxygen activation and ROS generation (114).

### Singlet Oxygen-Oriented Photodynamic Therapy

Taking all the above, we here propose the development of a novel therapy targeting singlet oxygen using photodynamic techniques. The expected outcome is ^1^O_2_ or other ROS-induced cancer cells apoptosis and tumor regression as nicely reported in recent studies by the mean of extracellular singlet oxygen generation (115, 116). To do so, we propose that photosensitizers such as methylene blue (MB), chlorophyll, and protoporphyrin could play an intermediary role in the electron decongestion of ETC by catalyzing the activation of ^3^O_2_ into ^1^O_2_ and thus promoting apoptosis by accumulation of ROS species (Figure 6B). All these photosensitizers could be highly excited with a light source at specific wave lengths. MB has a double application. It is used as a dye in the textile industry, as well as a medicine for its antimicrobial properties and also applied as an antidote during cyanide, inhibitor of the complex IV (CIV), poisoning, or in cases of methemoglobinemia (117). Therefore, when administered MB acts as a CIV in the reduction of dioxygen to the water molecules (118) (Figure 6B). This singlet oxygen-oriented photodynamic therapy (PDT) is thought as a mimicry to chemotherapies such as does animals with predators (113, 119, 120). In addition, we propose that ^1^O_2_-oriented PDT could increase sensitivity of tumor cells, specially the resistant and ones, to conventional therapies. Furthermore, our recent study in Chinese Hamster Ovary (CHO) cells supports this hypothesis. Indeed, we have reported that combination of MB and METABLOC reduced the Warburg effect in CHO and optimized monoclonal antibody (mAB) production, which is a marker of an increased mitochondrial OXPHOS (121). Finally, we strongly believe there are experimental evidences that the resistance of tumor to conventional treatments may be overcome by targeting cancer cells' “Achilles' heel,” the ROS accumulation, by introducing photosensitizers as “trojan horses.”

## Conclusion

Mitochondria are much more than just a factory for producing energy for the cell. It is a cornerstone between the three essential processes for maintaining the stability of a multicellular organism: proliferation, differentiation, and cell death. Poor regulation of asymmetric cell proliferation often leads to local destabilization of tissues and degenerates into tumor masses that escape all regulations by the immune system. Therapeutic approaches such as chemotherapies, metabolic therapies, immunotherapies, or even radiotherapy make it possible to eliminate the most fragile cancer cells. However, mitochondria confer the cells a great capacity for adaptation and resistance to these agents perceived as external stresses. Our proposals are a call to develop *soft skills* methods to *hijack* cancer cell metabolism. This involves the use of molecules easily ingested by cancer cells and perceived as beneficial to meet their energy demand. This is the case with photosensitizers like methylene blue, protoporphyrin or chlorophyll already implemented in plants. In the second step, one will be applying the photodynamic therapy in order to excite these molecules present in the cell. They will create highly reactive species of which singlet oxygen (^1^O_2_) seems to be the main produced at the level of the CI, CIII, and perhaps CIV complexes of the ETC. We speculate that the accumulation of ^1^O_2_ in mitochondria will trigger apoptosis in cancer cells, especially those that are resistant to conventional treatments.

## Author Contributions

JV wrote the manuscript and draw the figures. LS and MJ contributed to the thinking and reviewed the manuscript. All authors contributed to the article and approved the submitted version.

## Conflict of Interest

The authors declare that the research was conducted in the absence of any commercial or financial relationships that could be construed as a potential conflict of interest.
